# Accuracy and Precision of Consumer-Grade Wearable Activity Monitors for Assessing Time Spent in Sedentary Behavior in Children and Adolescents: Systematic Review

**DOI:** 10.2196/37547

**Published:** 2022-08-09

**Authors:** Antonio Martinko, Josip Karuc, Petra Jurić, Hrvoje Podnar, Maroje Sorić

**Affiliations:** 1 Faculty of Kinesiology University of Zagreb Zagreb Croatia; 2 Proprio Centre Physical Rehabilitation Centre Zadar Croatia; 3 Faculty of Sport University of Ljubljana Ljubljana Slovenia

**Keywords:** accuracy, precision, sedentary behavior, children, adolescents, wearable activity monitor, eHealth, digital health, mobile health, mHealth, mobile phone

## Abstract

**Background:**

A large number of wearable activity monitor models are released and used each year by consumers and researchers. As more studies are being carried out on children and adolescents in terms of sedentary behavior (SB) assessment, knowledge about accurate and precise monitoring devices becomes increasingly important.

**Objective:**

The main aim of this systematic review was to investigate and communicate findings on the accuracy and precision of consumer-grade physical activity monitors in assessing the time spent in SB in children and adolescents.

**Methods:**

Searches of PubMed (MEDLINE), Scopus, SPORTDiscus (full text), ProQuest, Open Access Theses and Dissertations, DART Europe E-theses Portal, and Networked Digital Library of Theses and Dissertations electronic databases were performed. All relevant studies that compared different types of consumer-grade monitors using a comparison method in the assessment of SB, published in European languages from 2015 onward were considered for inclusion. The risk of bias was estimated using Consensus-Based Standards for the Selection of Health Status Measurement Instruments. For enabling comparisons of accuracy measures within the studied outcome domain, measurement accuracy interpretation was based on group mean or percentage error values and 90% CI. Acceptable limits were predefined as –10% to +10% error in controlled and free-living settings. For determining the number of studies with group error percentages that fall within or outside one of the sides from previously defined acceptable limits, two 1-sided tests of equivalence were carried out, and the direction of measurement error was examined.

**Results:**

A total of 8 studies complied with the predefined inclusion criteria, and 3 studies provided acceptable data for quantitative analyses. In terms of the presented accuracy comparisons, 14 were subsequently identified, with 6 of these comparisons being acceptable in terms of quantitative analysis. The results of the Cochran *Q* test indicated that the included studies did not share a common effect size (Q_5_=82.86; *P*<.001). *I*^2^, which represents the percentage of total variation across studies due to heterogeneity, amounted to 94%. The summary effect size based on the random effects model was not statistically significant (effect size=14.36, SE 12.04, 90% CI −5.45 to 34.17; *P*=.23). According to the equivalence test results, consumer-grade physical activity monitors did not generate equivalent estimates of SB in relation to the comparison methods. Majority of the studies (3/7, 43%) that reported the mean absolute percentage errors have reported values of <30%.

**Conclusions:**

This is the first study that has attempted to synthesize available evidence on the accuracy and precision of consumer-grade physical activity monitors in measuring SB in children and adolescents. We found very few studies on the accuracy and almost no evidence on the precision of wearable activity monitors. The presented results highlight the large heterogeneity in this area of research.

**Trial Registration:**

PROSPERO CRD42021251922; https://www.crd.york.ac.uk/prospero/display_record.php?RecordID=251922

## Introduction

### Background

Wearable devices are part of a growing market and are trending in terms of monitoring physical activity (PA) and sleep. Widely attainable wearable activity monitors (WAMs) have a high demand, which is supported by projections of market size growth by the year 2028, with extrapolated values of US $138.7 billion being extrapolated [1]. In addition, the magnification of health problems related to sedentary lifestyles is expected to increase the demand for these types of products. In addition, the COVID-19 pandemic has added consciousness regarding an overall picture of fitness and health in the general public. Activity monitors function as a means of providing feedback to users, while also offering behavior change tools, tracking of progress, and data storage. Daily self-monitoring is a core component of WAMs, in addition to comparing results with those of other users, which could increase PA levels in the long term [2].

Consumer-based WAMs can be wrist-worn or attached to a piece of clothing on different parts of the body, such as the hip. Currently, WAMs generally use a triaxial accelerometer to capture bodily movement in 3 dimensions. The collected data are then analyzed by proprietary algorithms to estimate the daily number of steps, amount of expended energy, sleep quantity and quality, and time spent on activities of different intensities [3]. Although WAMs are directed toward and mostly used by consumers who are already conscious about their health and PA, these devices could also be used as measurement tools among researchers in the fields of health promotion and PA [2,4-6].

Research focused on technology (ie, accuracy and precision) is of great interest, whereas studies of WAMs in the context of treatment and in medical settings have also been increasing. A recent systematic review [7] that analyzed 463 studies demonstrated a significant growth rate in the annual number of publications that included WAMs between 2013 and 2017. Measurement accuracy is a vital consideration, as WAMs are frequently used as a tool in research and a way of advising health care decisions. Studies in this field of research rely on accurate and precise instruments with small errors to elucidate complex research questions in which measurement error limits statistical power [8]. Consumer-grade WAMs were deemed accurate when measuring heart rate and steps [3]. However, accuracy is susceptible to variation when different manufacturers and types of devices are considered, with lower accuracy being reported for sleep, distance covered, and time spent in different PA levels [5,6]. Regarding precision, it was reported that there is high precision among devices for steps, distance, expended energy, and sleep [5]. In contrast to the large number of already available and emerging WAMs, there is still limited evidence on the accuracy and precision of consumer-grade WAMs. Moreover, most of the limited evidence at present refers to the measurement of PA, whereas research on sedentary behavior (SB) is lacking. This also goes against the growing popularity of monitoring training, successive recovery, and components of individuals’ anthropological status with this type of technology [9]. When discussing the interactions of children with WAMs, contrasting research findings have been found, where some studies suggest that WAMs could be used to increase PA levels, and others have reported that WAM use over prolonged periods declines over time among children and adolescents [7]. Studies on WAM feasibility in children have shown that design, feedback features, and comfort while wearing the device were the most important factors [10].

Previously registered trials and conducted studies have most commonly identified the number of steps taken as the outcome of interest, followed by time spent in activity, sleep, energy expenditure, and distance covered as some other outcomes [7,11]. As recommendations have been provided for the first time by the World Health Organization on the associations between SB and health outcomes [12], it seems that research on SB will gain greater interest in the future. SB relates to low-intensity activities (<1.5 metabolic equivalents of tasks) and includes several bodily positions, such as lying, sitting, and reclining. SB is accompanied by a set of adverse health outcomes, and this association is already apparent in childhood [13]. Therefore, accurate, precise, and low-cost methods for measuring SB in children are important SB. Consumer-based WAMs could be of assistance in terms of reducing the financial costs and time spent by professionals when providing support and guidance for behavior change in children and adolescents. Different issues may arise when measuring SB, such as the following: (1) WAM placement; (2) how nonwear time is defined, epochs, and cutoff points; (3) setting the criteria for SB bouts and breaks; and (4) a combination of posture and motion data [14]. Although several systematic reviews have attempted to synthesize evidence on the accuracy of WAMs in measuring PA [5,6,15,16], similar studies related to SB are not available.

### Objectives

The main aim of this systematic review was to analyze the evidence available on the accuracy and precision of consumer-grade WAMs in assessing the time spent on any type of SB in children and adolescents.

## Methods

### Search Strategy

The search strategy followed the PRISMA (Preferred Reporting Items for Systematic Reviews and Meta-Analyses) guidelines [17]. The review protocol was registered with PROSPERO, an international prospective register of systematic reviews (CRD42021251922). Electronic databases PubMed (MEDLINE), Scopus, and SPORTDiscus (full text) were searched to find all relevant studies; in addition, ProQuest, Open Access Theses and Dissertations, Dart Europe E-Theses Portal, and Networked Digital Library of Theses and Dissertations electronic databases were searched as alternative literature sources of possible gray literature [18]. For each electronic database, a modified search strategy concerning specific and controlled vocabulary was used, with a variation of the following terms: *(children, adolescent, teen, youth) AND (fitness tracker, physical fitness tracker, activity monitor, activity tracker, wearable device, wearable) AND (sedentary behavior, sedentary, sedentary lifestyle, physical inactivity, sedentary time, rest, sitting position, reclining)*. A filter covering studies published in European languages was applied, and a time frame was set for studies ranging from January 1, 2015, to the day of this systematic review’s execution, April 15, 2021. The term European languages refers primarily to some of the most commonly spoken languages in Europe, namely English, German, French, Italian, and Spanish, as the authors can understand these languages; therefore, the search was not limited only to studies in English. In addition, reference lists of the included studies and secondary sources were examined to find additional studies that were acceptable for inclusion. The constructed search strategy is presented in Multimedia Appendix 1.

### Study Selection and Eligibility Criteria

All papers retrieved from the electronic databases were gathered and organized into the Rayyan web application (Qatar Computing Research Institute, Hamad Bin Khalifa University) for systematic reviews [19]. Rayyan was used to screen for potential duplicates; a manual inspection was then performed to discover any additional duplicates. Titles and abstracts of the first 10% of the results were screened independently by 2 reviewers (AM and JK). Given that the interrater agreement was 100%, only one of the authors (AM) continued with the screening process for the remaining 90% of the results. In case of ambiguities, the authors resolved the situation through a discussion with a third reviewer (MS). After the initial screening, full texts of the selected studies were accessed and screened for eligibility by 2 independent reviewers (AM and JK). When needed, disagreements among reviewers were resolved through a discussion with the third reviewer (MS).

The search was limited to studies that involved participants aged <18 years, namely children and adolescents. Studies that included participants with a physical disability or any other condition precluding them from engaging in PA were excluded. Inclusion was possible for studies conducted in both controlled and free-living settings. Studies were considered for inclusion only when the accuracy of a consumer-grade PA monitor was examined in comparison with an appropriate research-grade device in relation to a specific study setting. Appropriate research-grade devices were predefined and included indirect calorimetry, direct observation, and accelerometers. The main outcome of the eligible studies was the duration of SB, which relates to activities that do not increase energy expenditure substantially above the resting level and includes activities performed in a sitting or lying down position. Studies that were not available in full text were excluded as were studies that reported energy expenditure as their only outcome. During the literature search stage, no restrictions were set in terms of study type, although only original scientific papers were considered for inclusion, and secondary sources were excluded after their reference sections were manually inspected. Studies comparing different types of consumer-grade monitors without including a research-grade comparison method were also excluded. Data from one of the studies [20] were sought from the study authors, but no response was received across several modes of communication. Accuracy metrics labeled as acceptable for quantitative analyses were as follows: mean absolute percentage error (MAPE), standardized regression coefficient, odds ratio, correlation statistics, average error, limits of agreement, area under the curve or % sensitivity, % specificity or % positive predictive value), % negative predictive value, and likelihood ratio.

### Risk of Bias Assessment

All included studies were assessed for the risk of bias by AM. In case of uncertainty, a discussion with a second reviewer (MS) was required to reach a decision. For assessing the risk of bias, a Consensus-Based Standards for the Selection of Health Status Measurement Instruments (COSMIN) tool was used [21]. COSMIN is a checklist used to evaluate the methodological quality of included studies when conducting systematic reviews of measurement characteristics. Each aspect of methodological quality evaluation was appraised based on a proposed scoring system [22]; it could be either of excellent, good, fair, or poor quality. In line with previous studies [11], we used a modified checklist in which the assessment included 6 components relevant to our research aim. The design or methodology components focused on the following: (1) percentages of missing data, (2) missing data management, (3) adequate sample sizes, (4) acceptable criterion comparisons, (5) design or methodological flaws, and (6) reporting of acceptable accuracy metrics, the only analytical component.

### Data Extraction and Coding

Study characteristics and outcomes were extracted by a single reviewer (PJ), whereas cross-checking of the table was performed by a second reviewer (AM) familiar with the details of the included studies. Potential conflicts were resolved through discussion with a senior reviewer (MS). The extracted data included the reference, study period, participants (number, age, sex, ethnicity, socioeconomic status, and inclusion and exclusion criteria), type of consumer-grade PA monitor, comparison method, context of SB (ie, setting and type of activity), cutoff points for SB, and reported accuracy metrics. Regarding accuracy metrics, MAPE, standardized regression coefficients, odds ratio, correlation statistics, average error, limits of agreement, area under the curve, % sensitivity, % specificity, % positive predictive value, % negative predictive value, and likelihood ratio were extracted if available. In cases where group percentage differences were not reported in the study, a group percentage error was calculated ([Consumer-grade_mean_ – Research-grade_mean_] / Research-grade_mean_ × 100). This was performed to acquire a common unit of measurement for enabling comparisons of accuracy measures within the studied outcome domain [6,11]. Except for % differences (ie, errors), 95% CIs were extracted if they were reported or calculated if appropriate data were accessible.

### Statistical Analysis

All quantitative investigations were carried out in RStudio (version 4.1.2) [23], using the meta [24], metafor [25], TOSTER [26], gridExtra [27], dmetar [28], and ggplot2 [29] packages for producing the results and plots of the meta-analysis. A random effects model was used to pool the effect sizes and SEs of the included studies. Two 1-sided tests of equivalence were carried out to determine the number of studies with group error percentages that fall within or outside one of the sides from previously defined acceptable limits and the direction of measurement error was examined. Studies with large sample sizes conducting difference tests are more likely to find statistically significant differences, whereas studies with smaller sample sizes are less likely to do so; both cases lead to incorrect conclusions [8,26]. Although tests of mean difference are a common statistical approach in measurement agreement research, equivalence testing was developed to provide *evidence of equivalence* directly, in contrast with inferring *no evidence of differences* among different devices [8]. Two 1-sided tests of equivalence were conducted to compare the 90% CIs of the estimates from the consumer-grade PA monitors with the defined equivalence zone (EZ) extrapolated from the comparison method. Although no formal guidelines exist to define the best EZ, the interpretation of measurement accuracy in this study included predefined acceptable limits for measurement accuracy of –10% to +10% in controlled and free-living settings, in line with previous secondary publications [6,11] and based on a series of previous primary publications [30-32]. Testing for whether the 90% CI from the measurements of consumer-grade PA monitors falls within the determined EZ was conducted with a statistical significance set at .05.

Because of the variability in the consumer-grade and research-grade devices and the methods used, heterogeneity was suspected; therefore, the random effects model was chosen. Cochran *Q* test and *I*^2^ test were used to assess heterogeneity and the degree of inconsistency, respectively, across studies [33,34]. Both tests were used, because significant heterogeneity among studies was poorly detected by the Cochran *Q* test when a small number of studies was included in the meta-analysis, as the power of the test is low under such conditions [33]. *I*^2^ was used, as it represents the percentage of total variation across studies that is because of heterogeneity, ranging between 0% and 100%, where values of 25%, 50%, and 75% point to low, moderate, and high heterogeneity, respectively [33]. As noted earlier, some heterogeneity in the true effect sizes among studies was expected. Outlier and influence analyses were performed to investigate the causes of these problems. As several methods are present for determining outliers in meta-analyses [35], the dmetar package [28] in R contains a *find.outliers* function, which attempts to identify outlying studies included in the meta-analysis, after which these studies are removed, and the pooled effects are recalculated. Influential studies also have a substantial effect on the pooled effect or heterogeneity, and techniques used to identify these studies are based on the leave-one-out method [35]. In the leave-one-out method, recalculation of the results is performed as many times as there are included studies in the meta-analysis, leaving out one study each time [35]. Using the dmetar package [28] and an accompanying *InfluenceAnalysis* function, various influence diagnostics were calculated. A Baujat plot was constructed to illustrate studies that influence overall heterogeneity and the overall result, where those that fall to the top right quadrant have the most influence [36]. Two forest plots of the leave-one-out meta-analyses were also constructed: the first one being sorted by the pooled effect size and the second by the *I^2^* value. Effect estimates plotted against sample sizes used in the studies were visually inspected with funnel plots, where publication bias and other biases were identified if a skewed and asymmetrical plot was present [37].

Alternatively, a narrative synthesis was performed for accuracy analyses that did not report data, allowing for the inspection of group percentage errors. Furthermore, the consistency of accuracy metrics available from those studies with the quantitative synthesis was narratively outlined, in addition to the direction of the measurement error.

## Results

### Study Selection

**Figure 1:**
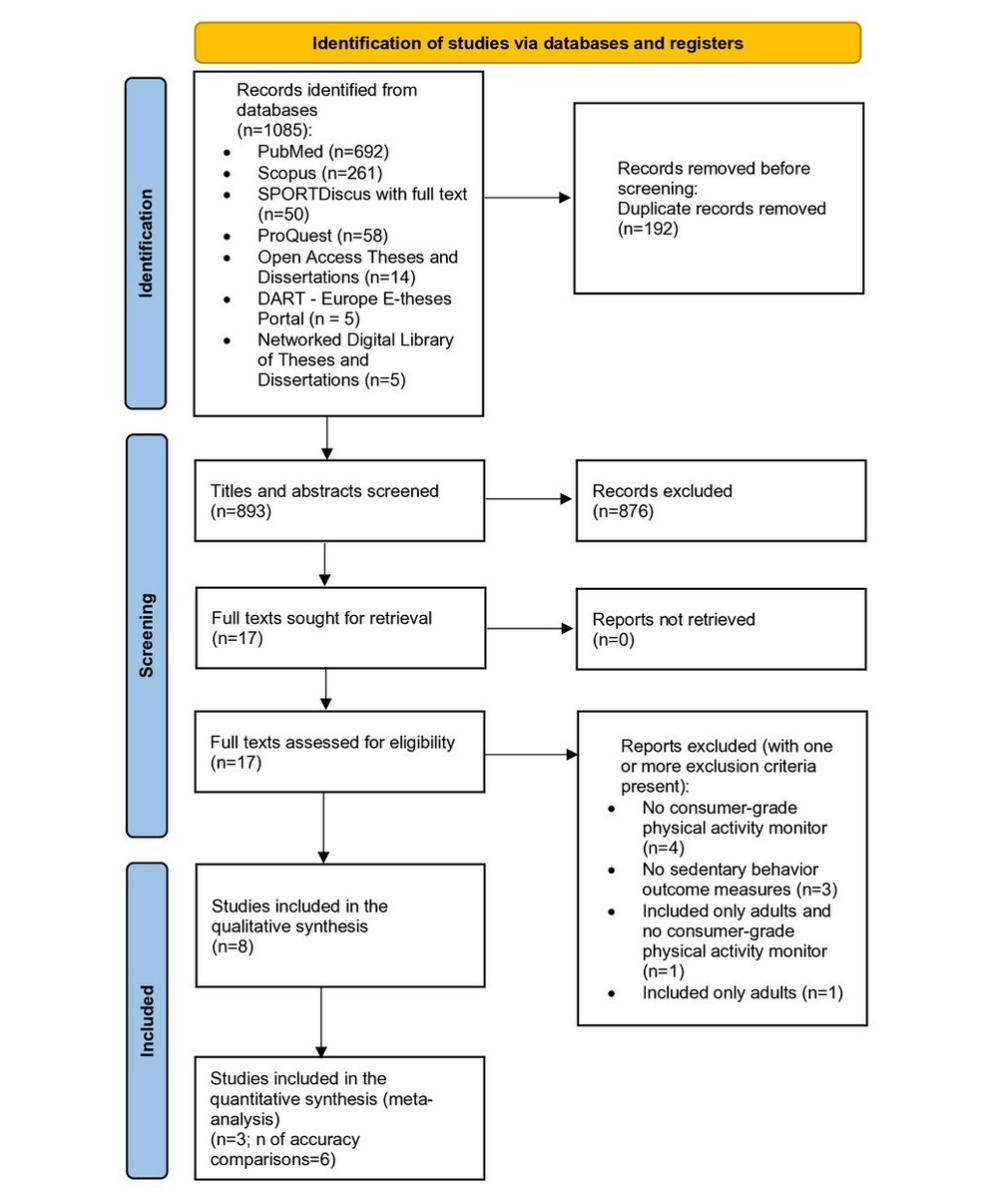
PRISMA (Preferred Reporting Items for Systematic Reviews and Meta-Analyses) flowchart.

After applying our search strategy to designated electronic databases, 1085 studies were identified. Further, duplicates were removed, and 82.3% (893/1085) of titles and abstracts were carried over to screening for eligibility. The exclusion of 98.1% (876/1085) of studies left us with 1.9% (17/1085) of studies in which full-text screening was conducted. Following the full-text screening, 9 additional studies were excluded if at least one of the following reasons were present: no consumer-grade PA monitors were used (7/9, 78%), included only adults (2/9, 22%), and no SB outcome measures were assessed (2/9, 22%). Finally, 8 studies complied with all predefined inclusion criteria, and 3 (38%) of these studies provided acceptable data for inclusion in the quantitative analyses. Only studies in English were found and deemed eligible, even though the search was not limited only to this language. The steps taken to identify the studies included in this review are detailed in Figure 1.

### Study Characteristics

All the information regarding study characteristics is summarized in Table 1. Of the included studies, 8 contained a total of 392 participants, whereas the mean number of participants was 49 (SD 41.36; range 10 to 144). Of the total number of participants, 195 were female (49.7%). Because one of the studies [38] included individuals aged 16 to 25 years with a median age of 19.3 (IQR 17-21) years, it was excluded from the participants’ mean age calculation. Hence, the mean age of participants was 8.3 (SD 2.39) years, with the age ranging from 4.8 to 10.3 years. Participants in all studies were healthy, except for one study, which included youth with mental health problems [38] but that did not preclude these participants from engaging in PA. Of the 8 studies, 4 (50%) were conducted in free-living settings [20,38-40], 3 (38%) studies were conducted in controlled settings [41-43], and 1 (13%) study was conducted in both controlled and free-living settings [44]. Most studies (6/8, 75%) used Fitbit devices (Fitbit Inc) as a consumer-grade WAM [20,38,39,41-43]. Of the 8 studies, the 2 (25%) remaining studies used the Polar active watch (Polar Electro Oy) [40] and Movband, Sqord, and Zamzee [44]. ActiGraph GT3X + (ActiGraph Inc) was most used as a comparison device in free-living settings [20,39,40], with both ActiGraph GT9X [40] and Actiwatch-64 (Philips Respironics) [38] being used in one study. In controlled settings, WAMs were compared with ActiGraph GT3X+ in 2 studies [42,44], whereas direct observation [41] and a portable indirect calorimeter (Cosmed K4B2; Cosmed Inc) [43] were used in one study each. Consumer-grade WAMs were worn on the nondominant wrist (6/8, 75%), dominant wrist (2/8, 25%), or the hip (2/8, 25%). Research-grade PA monitors were worn on the hip and attached to a belt on all the occasions. In studies conducted in free-living settings, children wore the WAM for 24 hours, whereas the duration of the monitoring period ranged from 1 to 7 consecutive days, although in one of the studies, children were observed across 5 days but only during an afterschool program that lasted for 80 minutes [40]. In studies conducted in controlled settings, sets of numerous unstructured or structured activities ranging in intensity are usually performed. The number and duration of these activities were similar, whereas the types of sedentary activity were also similar across studies and included sitting or lying while being quiet, watching television, listening to music, or playing video games. Across the included studies, some differences in the cutoff points used for identifying time spent in SB were present in both consumer-grade and research-grade PA monitors. For studies conducted in free-living settings, cutoff points for identifying SB were reported only in terms of research-grade PA monitors, and SB was usually equivalent to ≤25 counts per 15-second epoch or ≤100 counts per minute. A pair of studies conducted in free-living settings used 2 different cutoff points: SB <2.0 metabolic equivalent of tasks (METs) or <1.5 METs [40], and SB <37.5 counts or ≤25 counts per 15-second epoch. Most of the studies (3/4, 75%) conducted in controlled settings used similar cutoff points, that is, activities with MET values of 1.4 or <1.5 METs were identified as sedentary. Two of the studies did not report cutoff points for consumer- or research-grade PA monitors [38,44].

**Table 1:** Summary of study characteristics included in the systematic review (alphabetically by author and divided by study setting).

Author, year		Setting	Participants (female), n (%)		Age of participants (years), mean (SD)	Context of sedentary behavior (duration and type)	Type of device (body placement)		Cutoff point for sedentary behavior	Accuracy metric reported
							Consumer-grade	Research-grade		
Byun et al [20], 2018	Free-living		27 (11)	4.9 (1.0)		2 consecutive days (24-hour period)	Fitbit Flex (nondominant wrist)	ActiGraph GT3X+ (right hip)	Pate cutoff: <37.5 counts per 15 s; Evenson cutoff: ≤25 counts per 15 s	Pearson product moment correlation coefficients (*r*); MAPE^a^
Kim and Lochbaum [40], 2018	Free-living		51 (32)	10.30 (0.9)		Up to 5 consecutive days (afterschool program for 80 minutes in a predesignated classroom)	Polar active watch (nondominant wrist)	ActiGraph GT3X+ (waist); ActiGraph GT9X (nondominant wrist)	<2.0 METs^b^; <1.5 METs; Evenson cutoff: ≤50 counts per 30 s; Chandler cutoff:^c^ <966 counts per 30 s	Pearson product moment correlation coefficients (*r*); MAPE; regression coefficients; mean ratios for equivalence tests; level of agreement
Mooses et al [39], 2018	Free-living		144 (72)	9-10 y		Only during school hours for 2 weeks (one in September and one in November 2016)	Fitbit zip (hip^d^)	ActiGraph GT3x-BT (waist)	Evenson cutoff: ≤100 counts per min (1-min epochs)	Spearman correlation (*r*); limits of agreement^e^
Scott et al [38], 2019	Free-living		10 (6)	Median 19.3 (IQR 17-21)		7 consecutive days and nights (24-hour period)—the proportion of sedentary time	Fitbit^f^ (nondominant wrist)	Actiwatch-64 (nondominant wrist)	—^g,h^	MAPE; level of agreement
Sirard et al [44], 2017	Controlled and free-living		Phase 2: 14 (7); phase 3: 16 (8)	Phase 2: 9.0 (2.0); phase 3: 8.6 (1.6)		Phase 2: 10 activities on 2 occasions (sedentary activity for 5 min)—sitting quietly; phase 3: 4 consecutive days (24-h period)	Movband (dominant wrist); Sqord (dominant wrist); Zamzee (right hip)	ActiGraph GT3X+ (right hip)	Evenson cutoff: ≤100 counts per min	Phase 2: spearman rho coefficients; phase 3: Spearman correlation (*r*)
Byun et al [41], 2018	Controlled		28 (13)	4.8 (1.0)		A total of 6 activities for 34 min (sedentary activity for 8 min with a 1 min rest between)—sedentary (watching television lying down for 4 min and watching television sitting on a couch for 4 min)	Fitbit Flex 1 (nondominant wrist)	Direct observation	<1.4 METs (Fitbit 1-min epochs; direct observation 5- to 15-s epochs)	Pearson correlation coefficients (*r*); MAPE; Cohen κ; sensitivity; specificity; ROC-AUC^i^
Godino et al [43], 2020	Controlled		59 (31)	9.9 (0.7)		14 activities for 2-3 h (each sedentary activity for 5 min)—sedentary (sitting quietly, listening to music, and playing games on iPad)	Fitbit Charge HR (nondominant wrist)	Cosmed K4B2 (fitted according to manufacturer recommendations)	<1.5 METs (1-min epochs)	MAPE; Cohen κ; sensitivity; specificity
Kang et al [42], 2019	Controlled		43 (18)	9.7 (1.3)		12 activities for 48 min (each 3 min with a 1 min rest between)—sedentary (sitting quietly in a chair, playing a video game, and watching television)	Fitbit Charge HR (dominant and nondominant wrist)^j^	ActiGraph GT3X+ (dominant and nondominant wrist)^j^	<1.4 METs (1-min epochs)	Pearson correlation coefficients (*r*); MAPE; Cohen κ; sensitivity; specificity; ROC-AUC; ICC^k^ (95% CI)

^a^MAPE: mean absolute percent error.

^b^MET: metabolic equivalent of task.

^c^Due to an error during production, Chandler cutoff points at the 30-second epoch length were incorrectly presented in the published paper (the corrected cutoff points are inserted in Table 1).

^d^The accelerometer and Fitbit Zip were attached on the hip with the same elastic belt and worn on the same side.

^e^Bland-Altman analysis with the calculation of bias between 2 devices (the mean of differences of the 2 devices).

^f^Model not reported.

^g^Not available.

^h^Sedentary behavior reported as a 0 to 1 value which represents the number of minutes sedentary divided by the morning time.

^i^ROC-AUC: area under the receiver operating curve.

^j^Random counterbalance of the wear position between the ActiGraph and Fitbit tracker on the wrist.

^k^ICC: intraclass correlation.

### Risk of Bias

The risk of bias assessment results are presented in Table 2. On the individual COSMIN component level, all included studies were rated as either excellent or good in 3 components of the methodological quality evaluation, relating to the reporting of missing data, handling missing data, and use of an adequate criterion comparator (ie, device). In terms of acceptable accuracy metrics, 75% (6/8) of studies were rated as excellent (n=4, 67%) or good (n=2, 33%), and 25% (2/8) of studies were rated as poor because no percentage error was reported or a way to calculate it was present, although the studies reported other measures of accuracy. Instead of entirely excluding these studies from the review, the reported measures of accuracy, and their consistency with the examination of percentage measurement error are narratively outlined. When examining the components related to important methodological flaws in the design or execution of the studies, of the 8 studies, 6 (75%) studies were rated excellent (n=5, 83%) or good (n=1, 17%), and 2 (25%) were rated fair. In contrast to the scoring of most COSMIN components, most studies were rated as fair (1/8, 13%) or poor (4/8, 50%) in the adequate sample size component. Regarding the studies, of 8 studies, only 1 (13%) study was rated excellent and 2 (25%) studies were rated good in terms of sufficient sample size. No studies were excluded from the analysis owing to poor methodological quality, as only the adequate sample size component was unfavorable. Only one study had more than 100 participants (N=144) and was rated as excellent; therefore, no restrictions were set in terms of inclusion for the minimum number of participants needed in a study.

**Table 2:** Results of risk of bias assessment for studies included in the systematic review (N=8)^a^.

Study details		Summary: excellent or good, n (%)	COSMIN^b^ risk of bias assessment					
Author, year published	Study setting		Reporting missing data	Handling missing data	Adequate sample size	Acceptable comparison	Flaws in design or methods	Acceptable accuracy metrics
Kim and Lochbaum [40], 2018	F^c^	6 (100)	Good	Good	Good	Excellent	Excellent	Excellent
Mooses et al [39], 2018	F	6 (100)	Excellent	Good	Excellent	Excellent	Excellent	Excellent
Byun et al [20], 2018	F	5 (83)	Good	Good	Poor	Excellent	Excellent	Good
Scott et al [38], 2019	F	4 (67)	Excellent	Excellent	Poor	Good	Fair	Excellent
Godino et al [43], 2020	C^d^	6 (100)	Excellent	Excellent	Good	Excellent	Good	Excellent
Byun et al [41], 2018	C	5 (83)	Good	Good	Poor	Good	Excellent	Good
Kang et al [42], 2019	C	4 (67)	Good	Good	Fair	Excellent	Excellent	Poor
Sirard et al [44], 2017	C, F	3 (50)	Excellent	Excellent	Poor	Excellent	Fair	Poor

^a^The summary of excellent or good values of reporting missing data, handling missing data, adequate sample size, acceptable comparison, flaws in design or methods, and acceptable accuracy metrics are 8 (100%), 8 (100%), 3 (38%), 8 (100%), 6 (75%), and 6 (75%), respectively.

^b^COSMIN: Consensus-Based Standards for the Selection of Health Status Measurement Instruments.

^c^F: free-living.

^d^C: controlled.

At the individual study level, the cutoff point for high study quality was set arbitrarily and was defined as scoring excellent or good on 88% (7/8) or 100% (8/8) of the components. In free-living settings, 3 studies were evaluated as being of high quality [20,39,40], whereas in controlled settings there were 2 studies of high quality [41,43]. All studies scored excellent or good on more than half of the COSMIN risk of bias components, although 3 of the studies could not be considered high quality [38,42,44]. The only study carried out in both free-living and controlled settings [44] had the lowest number of excellent or good scores (5/8, 62%) and also scored poorly on 2 components (ie, adequate sample size and acceptable accuracy metrics).

### Accuracy of Time in SB Measurements

#### Quantitative Synthesis

**Figure 2:**
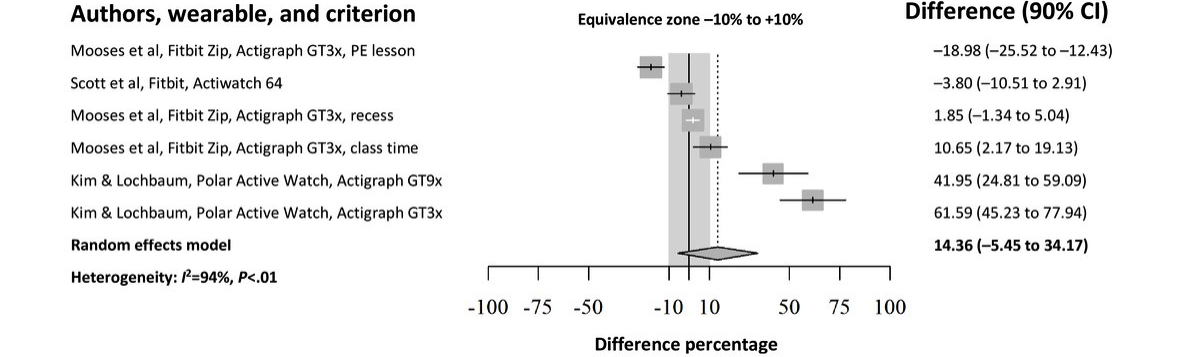
Forest plot of consumer-grade physical activity monitors accuracy in relation to research-grade monitors for assessing sedentary behavior in children. Squares represent point estimates, and 90% CIs are indicated by lines. The pooled effect size and 90% CI of the random effects model are shown at the bottom, represented by a diamond. The dashed line represents the predefined equivalence zone of the comparison method (ie, –10% to +10%). PE: physical education. Mooses et al [40] (activity: physical education lesson); Scott et al [39]; Mooses et al [40] (activity: recess and activity: class time); Kim and Lochbaum [41] (research-grade physical activity monitor: ActiGraph GT9X and research-grade physical activity monitor: ActiGraph GT3X+).

In total, 8 studies containing 14 accuracy comparisons examined the accuracy of consumer-grade PA monitors in relation to a comparison method in assessing time spent in SB in children, although out of the total number, only 3 (38%) studies reported acceptable data for inclusion in the quantitative analyses. From the 3 studies included in the quantitative synthesis, 6 comparisons were examined. The random effects model results as seen in Figure 2. provided an estimated model coefficient (ie, the summary effect size) of 14.4% (SE 12%, 90 % CI −5.5% to 34.2%; *P*=.23).

The equivalence test results showed that consumer-grade PA monitors did not generate equivalent estimates of SB compared with research-grade monitors. The overall effect with the corresponding 90% CI was not completely within the predefined EZ of the comparison method (ie, –10% to +10%). It is also important to note the direction of the overall effect and the corresponding 90% CI with regard to the defined EZ of the comparison method, where it is evident that consumer-grade PA monitors overestimated SB compared with research-grade devices. Point estimates and corresponding 90% CIs of only 1 out of 6 accuracy analysis were located inside the predefined EZ [38], with a 90% CI of one additional accuracy analysis being borderline equivalent [39].

The results of the Cochran *Q* test indicated that heterogeneity among the population effect sizes estimated by the individual studies was present (Q_5_=82.86; *P*<.001). Furthermore, the *I*^2^ statistic [33] was 94% (95% CI 90.4%-96.2%), indicating very large heterogeneity. Although the Q-statistic and *I*^2^ provide evidence regarding heterogeneity, there is no information on which studies may influence overall heterogeneity. The search for potential outlying accuracy analyses yielded 2 results [39,40]. After excluding the identified studies from the meta-analyses, *I*^2^ decreased, although only marginally, from 94% to 84.5%, and the *Q* test of heterogeneity was still significant (*P*<.001). In addition, by removing these 2 studies, the pooled estimates were brought closer to the defined EZ from 14.4% (90% CI −5.5% to 34.2%) to 10.8% (90% CI −4.1% to 25.7%). Leave-one-out meta-analyses were also conducted with visualization of the results through forest plots that are presented in Multimedia Appendix 2. In the leave-one-out meta-analysis sorted by the pooled effect size, we found that the overall % difference was the largest when we removed 1 of the 2 outlying and influential studies [39] that had a very high contribution to the pooled effect size. In the second leave-one-out meta-analysis sorted by the values of *I*^2^ (ie, heterogeneity), omitting one of the studies [40] led to the largest decrease in *I*^2^. In conclusion, the results of the outlier and influence analyses indicate that the 2 studies [39,40] are likely influential outliers. Hence, a sensitivity analysis was conducted, in which these studies were excluded. The changes in the pooled effect size, *I*^2^, and CIs associated with removing influential studies are shown in Table 3.

**Table 3:** Random effects model results before and after removing the outliers.

Analysis	Pooled effect size (%, 90% CI)	*P* value	*I*^2^ (%, 95% CI)
Main analysis	14.4 (−5.5 to 34.2)	.23	94 (90.4 to 96.2)
Influential studies removed^a^	10.8 (−4.1 to 25.7)	.23	84.5 (66.4 to 92.8)

^a^Studies removed as outliers: Mooses et al [39] (activity: physical education lesson) and Kim and Lochbaum [40] (research-grade physical activity monitor: ActiGraph GT3X+).

**Figure 3:**
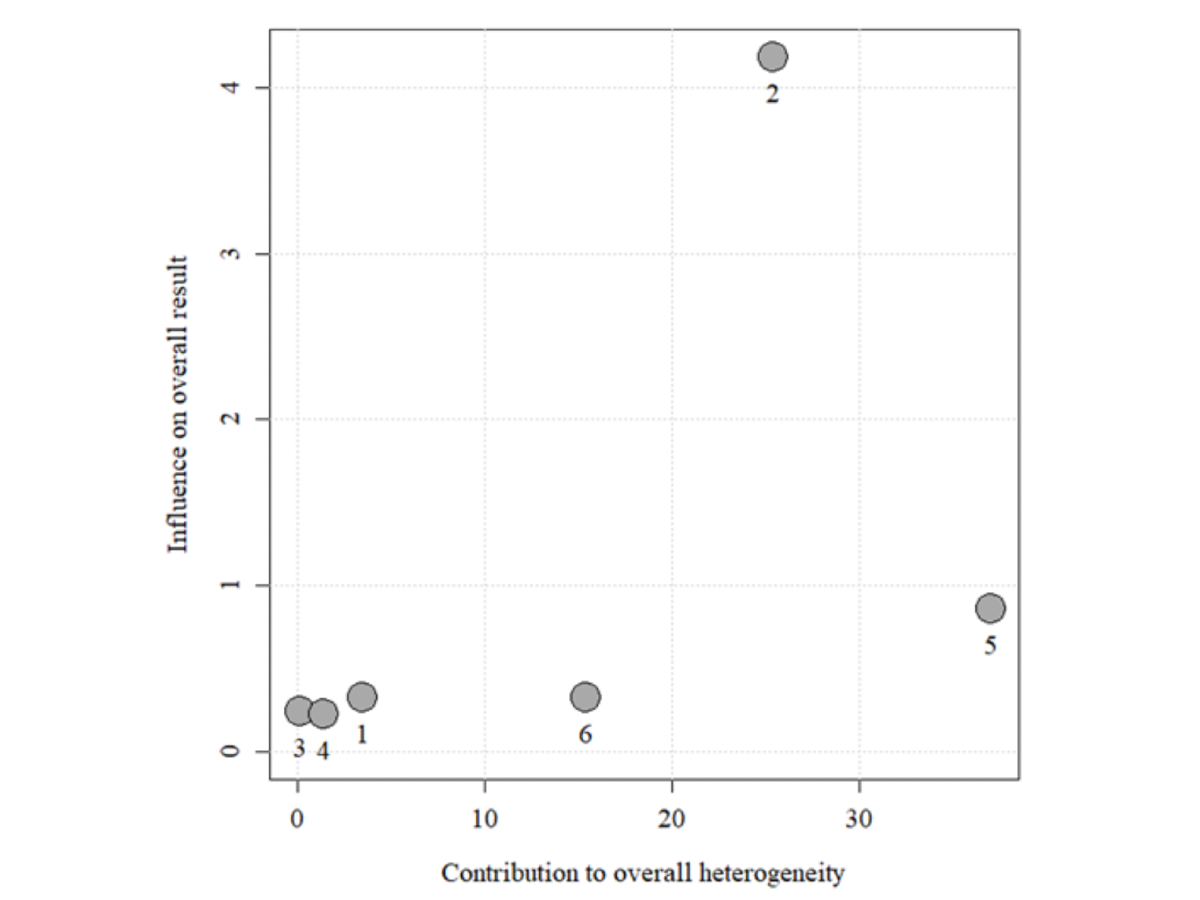
Baujat plot showing the influence of individual studies on the overall heterogeneity and the overall result where studies falling closer to the top right quadrant have the most influence. 1: Mooses et al [40] (activity: class time); 2: Mooses et al [40] (activity: physical education lesson); 3: Mooses et al [40] (activity: recess); 4: Scott et al [39]; 5: Kim and Lochbaum [41] (research-grade physical activity monitor: ActiGraph GT3X+); 6: Kim and Lochbaum [41] (research-grade physical activity monitor: ActiGraph GT9X).

As there is evidence of overall heterogeneity, the Baujat plot can display studies that contribute to overall heterogeneity and overall results [35]. A Baujat plot is shown in Figure 3. with the respective ID numbers used to differentiate the individual accuracy comparisons. Accuracy comparison [40] ID number 5 contributed the most to the overall result as well as the overall heterogeneity. Accuracy comparison [39] ID number 2 contributed the most to the overall heterogeneity and results, being closest to the upper right corner of the plot. A closer look at the characteristics of this accuracy comparison revealed that using different models and placements of consumer-grade and research-grade PA monitors could be potential moderating variables that may contribute to heterogeneity. In 2 identified outlying and influential accuracy analyses, one of the studies placed the consumer-grade PA monitor (ie, Fitbit Zip) at the hip [39], contrasting the placement in other included studies, whereas the other study used a Polar active watch as the consumer-grade PA monitor [40] also contrasting other studies.

A funnel plot was constructed to assess publication bias and is provided in Multimedia Appendix 3. Because of the low power of asymmetry statistical tests when <10 studies were included [45], only a visual inspection of the funnel plot was carried out. After visual inspection, an asymmetry in the plot was noticed, indicating the possibility of publication bias, which should not be equated with it, as several conceivable causes are plausible [37,45].

#### Narrative Synthesis

Results from the included accuracy analyses that did not report data that would allow for the quantitative analysis of the accuracy of consumer-grade PA monitors in assessing the time spent in SB in children are narratively outlined in the following sections. In these studies, the results pertaining to other available accuracy metrics are summarized. This narrative synthesis encompasses 5 studies, in which 8 accuracy analyses were identified. Of the total 8 studies, 2 (40%) studies were conducted in free-living settings, of which a study was also carried out in controlled settings in one of the implementation phases. Byun et al [20] reported a mean difference of 42 to 71 minutes per day during 2 consecutive days among the devices when measuring SB, where the 90% CI for the mean estimates from the consumer-grade PA monitor (ie, Fitbit Flex) was within 15% of the mean estimates from ActiGraph GT3X+. In addition, a strong correlation of the time spent in SB between the 2 devices has been reported (*r*=0.87) [20]. Sirard et al [44] also used ActiGraph GT3X+ as a research-grade PA monitor in controlled and free-living settings for 4 consecutive days to assess the accuracy of several WAMs and reported a high correlation in SB time assessed in free-living conditions for Movband (*r*=0.76) and Sqord (*r*=0.86) PA monitors, whereas a moderate correlation was reported for Zamzee (*r*=0.59). Of note for this study, in phase 2, which was conducted in controlled settings, all the devices differentiated SB from light-intensity PA with similar accuracy as the research-grade PA monitor [44]. Moving on now to consider controlled settings, the sensitivity and specificity of SB detection for the Fitbit Flex device reported by Byun et al [41] amounted to 96.8% and 88.6%, respectively, with high SB classification accuracy (90.2%) and high area under the receiver operating characteristic curve values (0.92). In this study, the Fitbit Flex produced a negligible bias in SB estimation, with approximately 2 more minutes of SB recorded in relation to the criterion method (ie, direct observation) [41]. High sensitivity (84.8%) and specificity (83.1%) values have also been reported by Godino et al for Fitbit Charge HR for classifying SB or light PA [43]. A similar performance of Fitbit Charge HR was recorded in the study by Kang et al [42] with a sensitivity of 91.6% and specificity of 72.4%. Values were consistently high for studies conducted in controlled settings, as also reported by Kang et al [42]. Herein, the classification accuracy (80.73%) and area under the receiver operating characteristic curve (0.82) values were also high for Fitbit Charge HR [42].

A common metric reported in most of the studies was MAPE, where considerable disagreement when measuring SB (ie, MAPE >60%) was present only in the study by Kim and Lochbaum [40]. Kim and Lochbaum [40] reported MAPEs of 121.68% (95% CI 84.87%-158.49%) and 122.73% (95% CI 53.9%-191.57%) for SB <2.0 METs but lower MAPEs of 69.92% (95% CI 63.39%-76.44%) and 79.84% (95% CI 55.21%-104.46%) for SB <1.5 METs when comparing Polar active watch with ActiGraph GT3X+ and ActiGraph GT9X, respectively. Hence, the results depended mostly on the defined SB cutoff points. Most studies that reported MAPEs reported values of <30% [20,38,41]. A total of 2 studies conducted in children of similar ages (4.9, SD 1.0 years and 4.8, SD 1.0 years) with the same consumer-grade PA monitor (Fitbit Flex) reported MAPEs of 9.1% and 13.0% based on different cutoff points [20] and 28.8% [41], respectively. Accordingly, differences appeared because the first study was conducted in free-living settings, comparing Fitbit Flex with a comparison device (ie, ActiGraph GT3X) [20], whereas the other study compared Fitbit in controlled settings with a criterion method (ie, direct observation) [41], where larger differences were expected. Undoubtedly, values depend mostly on the cutoff points used, settings or used devices. The study by Kim and Lochbaum [40] can be seen as an outlier in both quantitative synthesis and narrative synthesis. This disparity with other results could be due to the previously mentioned arguments revolving around differences in used devices, cutoff points, and settings. Not enough data were provided to try narratively synthesizing the direction of differences between consumer-grade and research-grade PA monitors. Regarding precision, no study has reported data on the precision of consumer-grade WAMs in assessing the time spent on any type of SB in children and adolescents. Only one of the included studies examined precision; however, SB was not considered an outcome in these analyses. In general, this study reported good precision for moderate to vigorous PA, energy expenditure, steps, and heart rate among devices carried on the wrists of both hands [42]. Furthermore, a study examined only interunit variability during orbital shaker testing, which is related to repeatability as one of the aspects of precision**.** Of the 3 devices used across a range of frequencies (1.3, 1.9, and 2.5 Hz), Movband showed the lowest interunit variability (coefficient of variation [CV] 0.62, 0.85, and 0.19, with respect to frequencies), whereas Sqord (CV 29.8, 3.85, and 1.93, respectively) and Zamzee produced worse results (CV 25.5, 12.1, and 9.75, respectively) [44]. These results could be relevant in situations when they are used in groups (eg, classrooms) where different children involved in the same activity may present different results in terms of the measured activity.

## Discussion

### Principal Findings

This review is one of the first studies that summarized data on the accuracy of consumer-grade PA monitors when measuring the time spent in any type of SB in children and adolescents. On the basis of the limited amount of data available for quantitative synthesis, it seems that consumer-grade PA monitors did not generate equivalent estimates of SB compared with research-grade monitors, with a tendency toward overestimation for these devices. In contrast, narrative synthesis suggested that at least some of these devices (ie, Fitbit) should be viewed as an accurate method of SB measurement in children and adolescents owing to the high levels of classification accuracy found in several individual studies.

The fact that WAMs were not found to be equivalent to research-grade monitors in this study should not be interpreted as having low accuracy. In all included studies conducted in free-living settings, accelerometers were used to determine the accuracy of consumer-grade PA monitors. Accelerometers cannot be regarded as the gold standard for measuring SB, although they produce results similar to a criterion method (ie, inclinometers). Accelerometers placed on the hip with their corresponding cutoff points overestimate the time spent in SB in comparison with a criterion method when young children, adolescents, and adults are considered because standing is also included as one of the inspected postures [14]. In general, criterion methods were only used in controlled settings in 25% (2/8) of the included studies. Controlled settings are appropriate for examinations of “genuine” accuracy although that does not necessarily translate to free-living settings, in which consumer-grade PA monitors are intended to be used. Comparing the accuracy of consumer-grade PA monitors in relation to criterion methods in free-living settings is difficult, where the use of these devices over several days when the participant is engaged in their everyday activities is not feasible*.* Therefore, it is uncertain whether the accuracy of consumer-grade PA monitors is poor when they are used to measure SB, especially in free-living settings. The specific aims of the study and the significance of accuracy and practicality should be considered when selecting a suitable device [46]. Using gold standards (eg, indirect calorimetry) could be needed in clinical studies; however, the cost and difficulties encountered with using them make them unsuitable for large samples located in free-living settings [47]. This problem is even more pronounced when working with children than with adults [47].

The random effects model results indicate that consumer-grade PA monitors overestimate the amount of time spent in SB, while removing 2 influential and outlying studies brought the estimates closer to the defined EZ. The placement of WAMs could be a potential moderating variable because Fitbit was placed in one of these studies at the hip [39], whereas some previous studies also reported overestimation of SB from hip-based accelerometers in adolescents and adults [14]. As overestimation of SB in our study was noticed for consumer-grade PA monitors mostly in relation to accelerometers, potentially even higher levels of overestimation would be present if the gold standard was used for comparison. A research-grade device measuring the inclination of the thigh, such as activPal, is regarded as accurate for SB measurement among children in free-living settings [48], because it uses an inclinometer, a sensor capable of better horizontal (sitting or lying) and vertical (standing) position classification [49]. The use of these types of sensors in consumer-grade PA monitors could potentially have positive effects on SB measurement accuracy. Therefore, placing the PA monitor on the thigh might also be suggested, as changes in thigh positions proved to be the most accurate way of measuring SB [49]. As the thigh is at different inclinations when sitting and standing, the future might offer alternative solutions if the identification of different positions and inclinations of the wrist when shifting from sitting to standing and engaging in PA could be developed in consumer-grade PA monitors [14,50]. As it was shown that PA monitors wear time, over a longer period, declines, and comfort was defined as one of the most important factors [10], wearing the device on the wrist could increase their acceptance among children and adolescents. A visual inspection of the plots showed that the study by Kim and Lochbaum [40] contributed the most to the levels of heterogeneity and pooled results. The authors used a Polar active watch as the consumer-grade PA monitor, unlike other studies that used Fitbit devices [40]. Differences in measurements of SB are represented by values of MAPE >60%, in contrast to other studies, and it seems that the Polar active watch in this case [40] is not an acceptable device for SB measurement. Placing the research focus mostly on one device brand (ie, Fitbit) and a couple of models (eg, Charge, Zip, and Flex) of that brand produces limited knowledge regarding the accuracy of consumer-grade PA monitors. This is why this fact is pointed out as a potential confounder, as excluding the study by Kim and Lochbaum [40] would certainly provide better results in terms of accuracy. The results of our study cannot be generalized to all consumer-grade PA monitors, as only a few brands have been analyzed to date. The discontinuation of certain models is inevitable as the market and interest grow, as well as technological development. Even though we only included studies published since 2015, most of the devices used in these studies have been discontinued (ie, Fitbit Charge HR, Flex, and Zip and Polar active watch), although companies still provide consumer support [51]. Advanced algorithms and sensors, such as inclinometers and heart rate monitors, typically present in current WAM models could provide more accuracy when measuring SB, and a large part of the devices included in this review did not contain any of them. Used only in studies conducted in controlled settings, the Fitbit Charge HR, which also contains a heart rate monitor, did not prove to be superior in terms of accuracy when compared with other consumer-grade PA monitors. This might be because the used algorithms, as Fitbit Charge HR is an older model when compared with other included Fitbit devices, even though it has multiple built-in sensors.

When consumer-grade PA monitors are used by children and adolescents, their accuracy in detecting SB might be affected because of the greater amount of time spent in postures not typically observed in adults (eg, crawling, squatting, and kneeling). A previous study reported that as children spent more time in previously mentioned postures than adults, an overestimation of time spent in SB recorded by the activPal was found [14]. The various epoch lengths reported in the studies included in this review may have contributed to conflicting results when assessing the accuracy of consumer-grade PA monitors in free-living and controlled settings. Epoch lengths used in studies conducted in free-living settings have generally been shorter (ie, 15 and 30 seconds) than those used in studies with controlled settings (ie, 1 minute). It is up to discussion whether shorter epochs are better at assessing SB compared with longer epochs in children and adolescents. When PA is considered, shorter epochs seem to be better because of children’s intermittent behavioral patterns [14,16]. However, in terms of SB, it is less likely that children will sit still for longer periods, which could partly explain the reported SB overestimation in free-living settings when shorter epochs were used. Applying longer epochs (ie, 1 minute) might result in underestimation of SB in children due to the sporadic nature of their movements, although no relevance of shorter SB epochs has been derived when it comes to impacts on the overall health [14].

Even if WAMs prove to be more accurate in assessing SB in the future, they may be limited by the fact that they do not recognize the context of SB. The context of SB is important, because higher durations or frequencies of screen time, television viewing, and video game use were previously mostly associated with a myriad of negative consequences (eg, body composition, cardiometabolic risk scores, physical fitness, and self-esteem) [52]. Although also defined as SB, more time spent reading and doing homework was associated with positive outcomes (eg, academic achievement) [52]. As limited data are available to discuss the precision of consumer-grade PA monitors, no specific discussion has focused on this issue. In future research, the precision of consumer-grade WAMs in assessing SB in children and adolescents should be considered. The characteristics of a good instrument emphasize both accuracy and precision, whereas the latter is neglected in this specific area of research.

High levels of heterogeneity were found in our study owing to differences in study protocols, type of wearable devices examined, comparison methods, sample sizes, and reported outcome measures, which complicated the analysis and comparisons among the results of the included studies. Although the risk of bias assessment showed high levels of methodological quality for all included studies and most acceptable accuracy metrics were reported, most included studies did not contain an adequate sample size. In line with our findings, it has recently been reported that studies evaluating data from wearable devices comprise different study designs with samples of varying characteristics and sizes, methodological approaches, devices used, and different cutoff points for activities across all intensities [9]. A recent review that included 23 validation studies of reported energy expenditure estimates from 58 devices comparing them to appropriate comparison devices suggested that most studies (87%) reported inappropriate accuracy indicators (eg, correlation coefficients) [53]. Sample sizes from the studies included in the review ranged from 13 to 60 participants (ie, 52% with sample sizes ranging from 20 to 30 participants). This agrees with our results that the sample sizes in this area of research are not adequate. Only half (52%) or even fewer studies reported the recommended accuracy metrics (ie, MAPE and equivalence test results) needed to evaluate the actual individual error [53]. Equivalence tests and difference tests depend on arbitrary levels of significance and sample sizes; therefore, MAPE seems to be the most appropriate accuracy metric [53]. The quantitative synthesis of the data in this study was complicated by the fact that not every accuracy metric provides the same information, which is a major problem in this area of research [53]. In studies conducted in free-living settings, WAMs were worn during the study course, lasting for 1 day and up to 7 consecutive days. According to a study by Trost et al [49], a monitoring period of 7 days provided optimal approximations of daily moderate to vigorous PA among children and adolescents. The only study trying to determine how many days of monitoring are needed to provide precise estimates of SB for children was conducted with preschoolers [54]. Precise estimates of the total daily time spent in SB were possible after 6 to 9 consecutive days of monitoring [54]. Hence, it is questionable whether the most commonly used period of 7 days of monitoring would be acceptable in terms of SB analysis in children and adolescents.

### Future Research

Regarding future research paths, the age and relevance of different consumer-grade devices and their models should be considered, as well as algorithms used, as they tend to constantly change with the growth of the accompanying market [1]. A large number of tested consumer-grade PA monitors are soon outdated or are no longer in use [55], adding to the complexity of this research area. In addition, Fitbit devices are the most commonly used as illustrated in this study and several other reviews [6,10,15,16,47], possibly because of their high market share and low cost. In contrast, no information on the accuracy of more expensive consumer-grade WAMs, such as smart- watches, in assessing SB is currently available. At the same time, several very low-cost WAMs are available on the market for prices as low as US $45.50 (eg, Mi Smart Band 6). These instruments provide an opportunity for mass PA promotion in children and adolescents, but their accuracy needs to be tested beforehand [55]. The transparency of the algorithms used by the devices and companies should be encouraged, because defining adequate wear time criteria and cutoff points for activities of different intensities is challenging at present. This is due to constant firmware updates, which are needed for further improvement of PA and SB measurement [11]. In addition to accuracy assessment, consumer-grade PA monitoring feasibility and acceptability research among children and adolescents is important. These types of studies are underrepresented in the literature, with results from a recent review showing that only approximately one-third of the studies (32%) investigated effectiveness, user engagement, and acceptability altogether [56]. In addition, descriptive statistics and visual analysis were performed in 60% of these studies when assessing effectiveness without using inferential statistics, and 18.9% of all studies had small sample sizes (ie, <13 participants) [56]. This could be of importance in terms of WAM acceptability among children, because the information from previous studies shows that one-third of consumer-grade PA monitor owners from the United States stopped using the device within 6 months of receiving the device, and just above 40% of them continued using it after 2 years have passed [14,50]. For children and adolescents, the definition of the epoch length that WAMs should use when measuring SB remains unresolved, and further research comparing the accuracy of consumer-grade with research-grade PA monitors conducted in free-living settings should be used to test the accuracy of different epochs [14]. Identification of contexts (ie, settings) in which examination of measurement properties has been previously conducted should be considered when choosing the appropriate device for examining SB in children and adolescents [15]. Hence, if the measurement properties of the selected tool are unknown in certain contexts, future research should also focus on examining the measurement properties of WAMs in these contexts to ensure certainty when these devices are applied outside the research settings [15]. Smartphones offer certain possibilities in this regard, as they could provide ways of context identification if data regarding screen time could be gathered and used in future research on SB. The rapid growth of the WAM market should be accompanied by additional validation studies, as the available evidence summarized in this study identified only a single study that has shown that consumer-grade PA monitors are comparable with research-grade devices in terms of SB measurement in children and adolescents. A caveat to consider is that this specific study included only 10 participants, and did not limit only to children and adolescents (median age 19.3, IQR 17-21 years) [38]. Also, all 10 participants reported depressive symptoms, 4 (40%) also reported anxiety symptoms, 3 (30%) hypomania symptoms and 1 (10%) had a history of hallucinations [38]. Therefore, conclusions regarding the accuracy of consumer-grade PA monitors for the entire childhood period cannot be drawn based solely on the results of this study.

### Strengths and Limitations

The strength of this study relates to the fact that a broad search of electronic databases was performed, which included searching for gray literature and manual searching of the included studies reference lists and secondary sources. Another significant strength of this study is that it is the first to examine the accuracy of consumer-grade PA monitors in assessing SB, encompassing a quantitative synthesis of the available data as well as a narrative synthesis of studies not suitable for meta-analysis. Limitations relate to the fact that during the time needed to complete all stages of this review, new studies could have been published, as this area of research is very dynamic. The consumer-grade PA monitoring market is volatile, with new models being constantly brought to the market, and the technology is continuously improving. Another minor limitation could be that during the study selection phase, only 1 reviewer screened 90% of the studies, although an interrater agreement of 100% was reached after the first 10% of the abstracts and titles were screened independently by 2 reviewers. A limitation related to the small number of primary studies included in this review should also be noted. Not including smartphone apps in the review limits the generalizability of our findings, as they also provide data related to the time spent on activities of different intensities. Smartphone apps are already in wide use among children; therefore, an examination of their accuracy in measuring SB should be performed in the future. Smart watches, which were not identified in any of the studies in this review, are also being accepted by children, although their price, battery life, and complex user interface represent certain disadvantages when used in this area of research with children [55]. None of the included studies used a smart watch to test the accuracy of SB measurement in children, and only smart bands were included. Studies lasting longer than 7 days were not included in this study as none have been identified, potentially serving as a limitation and a guide for future studies. Generally, at the individual component level, all included studies were either excellent or good in terms of missing data reporting, missing data handling, and use of an adequate comparison measure. However, most (5/8, 63%) of the studies consisted of small sample sizes (ie, <50), and the criterion method use was questionable, especially in controlled settings, as methods such as direct observation and indirect calorimetry were underrepresented.

### Conclusions

To our knowledge, this is the first review to focus specifically on the accuracy and precision of consumer-grade PA monitors when measuring SB in children and adolescents, but we found a small number of available studies, especially those suitable for conducting a meta-analysis. In the quantitative synthesis, no equivalence in the average time spent in SB was found when consumer-grade PA monitors were compared with research-grade monitors. High levels of heterogeneity were noted in the results, although point estimates and corresponding 90% CIs of only one individual study were located inside the predefined EZ, with a 90% CI of an additional accuracy analysis being borderline equivalent. Moreover, heterogeneity was discernible in terms of different study designs with samples of varying characteristics and sizes, methodological approaches, devices used, and differences in the cutoff points used when defining SB. The narrative synthesis suggests that consumer-grade PA monitors could be considered a valid method of SB measurement in children and adolescents. The results of our study will inform researchers, clinicians, and consumers on the measurement accuracy of widely attainable PA monitors when measuring SB in children and adolescents. However, more evidence is needed to reach robust conclusions about the accuracy and precision in measuring SB of children and adolescents, even for the most prevalent devices currently available on the market.

## Appendix

Multimedia Appendix 1 Search strategy.

Multimedia Appendix 2 Leave-one-out meta-analyses with accompanying forest plots.

Multimedia Appendix 3 Funnel plot.

## Abbreviations

COSMIN: Consensus-Based Standards for the Selection of Health Status Measurement Instruments
CV: coefficient of variation
EZ: equivalence zone
MAPE: mean absolute percentage error
MET: metabolic equivalent of task
PA: physical activity
PRISMA: Preferred Reporting Items for Systematic Reviews and Meta-Analyses
SB: sedentary behavior
WAM: wearable activity monitor

## References

[1] Grand View Research (2021). Fitness Tracker Market Size Worth $138.7 Billion By 2028 | CAGR: 18.9%: Grand View Research, Inc. CISION PR Newswire.

[2] Brickwood KJ, Watson G, O'Brien J, Williams AD (2019). Consumer-based wearable activity trackers increase physical activity participation: systematic review and meta-analysis. JMIR Mhealth Uhealth.

[3] Wright SP, Hall Brown TS, Collier SR, Sandberg K (2017). How consumer physical activity monitors could transform human physiology research. Am J Physiol Regul Integr Comp Physiol.

[4] Cheatham SW, Stull KR, Fantigrassi M, Motel I (2018). The efficacy of wearable activity tracking technology as part of a weight loss program: a systematic review. J Sports Med Phys Fitness.

[5] Evenson KR, Goto MM, Furberg RD (2015). Systematic review of the validity and reliability of consumer-wearable activity trackers. Int J Behav Nutr Phys Act.

[6] Fuller D, Colwell E, Low J, Orychock K, Tobin MA, Simango B, Buote R, Van Heerden D, Luan H, Cullen K, Slade L, Taylor NG (2020). Reliability and validity of commercially available wearable devices for measuring steps, energy expenditure, and heart rate: systematic review. JMIR Mhealth Uhealth.

[7] Shin G, Jarrahi M, Fei Y, Karami A, Gafinowitz N, Byun A, Lu X (2019). Wearable activity trackers, accuracy, adoption, acceptance and health impact: a systematic literature review. J Biomed Inform.

[8] Dixon PM, Saint-Maurice PF, Kim Y, Hibbing P, Bai Y, Welk GJ (2018). A primer on the use of equivalence testing for evaluating measurement agreement. Med Sci Sports Exerc.

[9] Düking P, Fuss FK, Holmberg HC, Sperlich B (2018). Recommendations for assessment of the reliability, sensitivity, and validity of data provided by wearable sensors designed for monitoring physical activity. JMIR Mhealth Uhealth.

[10] Ridgers ND, McNarry MA, Mackintosh KA (2016). Feasibility and effectiveness of using wearable activity trackers in youth: a systematic review. JMIR Mhealth Uhealth.

[11] Feehan LM, Geldman J, Sayre EC, Park C, Ezzat AM, Yoo JY, Hamilton CB, Li LC (2018). Accuracy of Fitbit devices: systematic review and narrative syntheses of quantitative data. JMIR Mhealth Uhealth.

[12] (2020). WHO 2020 guidelines on physical activity and sedentary behaviour. World Health Organization.

[13] (2020). WHO guidelines on physical activity and sedentary behaviour: web annex: evidence profiles. World Health Organization.

[14] Janssen X, Cliff DP (2015). Issues related to measuring and interpreting objectively measured sedentary behavior data. Meas Phys Educ Exerc Sci.

[15] Phillips SM, Summerbell C, Hobbs M, Hesketh KR, Saxena S, Muir C, Hillier-Brown FC (2021). A systematic review of the validity, reliability, and feasibility of measurement tools used to assess the physical activity and sedentary behaviour of pre-school aged children. Int J Behav Nutr Phys Act.

[16] Lynch BA, Kaufman T, Rajjo TI, Mohammed K, Kumar S, Murad M, Gentile NE, Koepp GA, McCrady-Spitzer SK, Levine JA (2019). Accuracy of accelerometers for measuring physical activity and levels of sedentary behavior in children: a systematic review. J Prim Care Community Health.

[17] Page MJ, McKenzie JE, Bossuyt PM, Boutron I, Hoffmann TC, Mulrow CD, Shamseer L, Tetzlaff JM, Akl EA, Brennan SE, Chou R, Glanville J, Grimshaw JM, Hróbjartsson A, Lalu MM, Li T, Loder EW, Mayo-Wilson E, McDonald S, McGuinness LA, Stewart LA, Thomas J, Tricco AC, Welch VA, Whiting P, Moher D (2021). The PRISMA 2020 statement: an updated guideline for reporting systematic reviews. BMJ.

[18] Phillips V, Barker E (2021). Systematic reviews: structure, form and content. J Perioper Pract.

[19] Ouzzani M, Hammady H, Fedorowicz Z, Elmagarmid A (2016). Rayyan-a Web and mobile app for systematic reviews. Syst Rev.

[20] Byun W, Kim Y, Brusseau T (2018). The use of a Fitbit device for assessing physical activity and sedentary behavior in preschoolers. J Pediatr.

[21] Mokkink LB, Terwee CB, Patrick DL, Alonso J, Stratford PW, Knol DL, Bouter LM, de Vet HC (2010). The COSMIN checklist for assessing the methodological quality of studies on measurement properties of health status measurement instruments: an international Delphi study. Qual Life Res.

[22] Terwee CB, Mokkink LB, Knol DL, Ostelo RW, Bouter LM, de Vet HC (2012). Rating the methodological quality in systematic reviews of studies on measurement properties: a scoring system for the COSMIN checklist. Qual Life Res.

[23] R Core Team (2021). R: a language and environment for statistical computing. R Foundation for Statistical Computing.

[24] Balduzzi S, Rücker G, Schwarzer G (2019). How to perform a meta-analysis with R: a practical tutorial. Evid Based Ment Health.

[25] Viechtbauer W (2010). Conducting meta-analyses in R with the metafor Package. J Stat Softw.

[26] Lakens D (2017). Equivalence tests: a practical primer for t tests, correlations, and meta-analyses. Soc Psychol Personal Sci.

[27] (2017). gridExtra: Miscellaneous Functions for "Grid" Graphics – R package version 2.3. The Comprehensive R Archive Network.

[28] Harrer M, Cuijpers P, Furukawa T, Ebert DD (2019). dmetar: Companion R Package For The Guide 'Doing Meta-Analysis in R'. R package version 0.0.9000.

[29] Wickham H (2009). ggplot2: Elegant Graphics for Data Analysis.

[30] Bai Y, Tompkins C, Gell N, Dione D, Zhang T, Byun W (2021). Comprehensive comparison of Apple Watch and Fitbit monitors in a free-living setting. PLoS One.

[31] Bai Y, Hibbing P, Mantis C, Welk GJ (2018). Comparative evaluation of heart rate-based monitors: Apple Watch vs Fitbit Charge HR. J Sports Sci.

[32] Bai Y, Welk GJ, Nam YH, Lee JA, Lee JM, Kim Y, Meier NF, Dixon PM (2016). Comparison of consumer and research monitors under semistructured settings. Med Sci Sports Exerc.

[33] Higgins JP, Thompson SG, Deeks JJ, Altman DG (2003). Measuring inconsistency in meta-analyses. BMJ.

[34] Alba AC, Alexander PE, Chang J, MacIsaac J, DeFry S, Guyatt GH (2016). High statistical heterogeneity is more frequent in meta-analysis of continuous than binary outcomes. J Clin Epidemiol.

[35] Viechtbauer W, Cheung MW (2010). Outlier and influence diagnostics for meta-analysis. Res Synth Methods.

[36] Baujat B, Mahé C, Pignon JP, Hill C (2002). A graphical method for exploring heterogeneity in meta-analyses: application to a meta-analysis of 65 trials. Stat Med.

[37] Egger M, Davey Smith G, Schneider M, Minder C (1997). Bias in meta-analysis detected by a simple, graphical test. BMJ.

[38] Scott J, Grierson A, Gehue L, Kallestad H, MacMillan I, Hickie I (2019). Can consumer grade activity devices replace research grade actiwatches in youth mental health settings?. Sleep Biol Rhythms.

[39] Mooses K, Oja M, Reisberg S, Vilo J, Kull M (2018). Validating Fitbit Zip for monitoring physical activity of children in school: a cross-sectional study. BMC Public Health.

[40] Kim Y, Lochbaum M (2018). Comparison of polar active watch and waist- and wrist-worn ActiGraph accelerometers for measuring children's physical activity levels during unstructured afterschool programs. Int J Environ Res Public Health.

[41] Byun W, Lee JM, Kim Y, Brusseau TA (2018). Classification accuracy of a wearable activity tracker for assessing sedentary behavior and physical activity in 3-5-year-old children. Int J Environ Res Public Health.

[42] Kang S, Kim Y, Byun W, Suk J, Lee JM (2019). Comparison of a wearable tracker with Actigraph for classifying physical activity intensity and heart rate in children. Int J Environ Res Public Health.

[43] Godino JG, Wing D, de Zambotti M, Baker FC, Bagot K, Inkelis S, Pautz C, Higgins M, Nichols J, Brumback T, Chevance G, Colrain IM, Patrick K, Tapert SF (2020). Performance of a commercial multi-sensor wearable (Fitbit Charge HR) in measuring physical activity and sleep in healthy children. PLoS One.

[44] Sirard JR, Masteller B, Freedson PS, Mendoza A, Hickey A (2017). Youth oriented activity trackers: comprehensive laboratory- and field-based validation. J Med Internet Res.

[45] Sterne JA, Sutton AJ, Ioannidis JP, Terrin N, Jones DR, Lau J, Carpenter J, Rücker G, Harbord RM, Schmid CH, Tetzlaff J, Deeks JJ, Peters J, Macaskill P, Schwarzer G, Duval S, Altman DG, Moher D, Higgins JP (2011). Recommendations for examining and interpreting funnel plot asymmetry in meta-analyses of randomised controlled trials. BMJ.

[46] Baranowski T, Simons-Morton BG (1991). Dietary and physical activity assessment in school-aged children: measurement issues. J Sch Health.

[47] Welk GJ, Corbin CB, Dale D (2000). Measurement issues in the assessment of physical activity in children. Res Q Exerc Sport.

[48] Aunger J, Wagnild J (2022). Objective and subjective measurement of sedentary behavior in human adults: a toolkit. Am J Hum Biol.

[49] Trost SG, Pate RR, Freedson PS, Sallis JF, Taylor WC (2000). Using objective physical activity measures with youth: how many days of monitoring are needed?. Med Sci Sports Exerc.

[50] Kang M, Rowe DA (2015). Issues and challenges in sedentary behavior measurement. Meas Phys Educ Exerc Sci.

[51] (2019). Fitbit kills Alta, Alta HR, Flex 2, and Zip. VentureBeat.

[52] Carson V, Hunter S, Kuzik N, Gray CE, Poitras VJ, Chaput JP, Saunders TJ, Katzmarzyk PT, Okely AD, Connor Gorber S, Kho ME, Sampson M, Lee H, Tremblay MS (2016). Systematic review of sedentary behaviour and health indicators in school-aged children and youth: an update. Appl Physiol Nutr Metab.

[53] Welk GJ, Bai Y, Lee JM, Godino J, Saint-Maurice PF, Carr L (2019). Standardizing analytic methods and reporting in activity monitor validation studies. Med Sci Sports Exerc.

[54] Byun W, Beets MW, Pate RR (2015). Sedentary behavior in preschoolers: how many days of accelerometer monitoring is needed?. Int J Environ Res Public Health.

[55] Henriksen A, Haugen Mikalsen M, Woldaregay AZ, Muzny M, Hartvigsen G, Hopstock LA, Grimsgaard S (2018). Using fitness trackers and smartwatches to measure physical activity in research: analysis of consumer wrist-worn wearables. J Med Internet Res.

[56] McCallum C, Rooksby J, Gray CM (2018). Evaluating the impact of physical activity apps and wearables: interdisciplinary review. JMIR Mhealth Uhealth.

