# Supplementary material for: Accuracy and Precision of Consumer-Grade Wearable Activity Monitors for Assessing Time Spent in Sedentary Behavior in Children and Adolescents: Systematic Review
Source: JMIR Mhealth Uhealth. 2022 Aug 9;10(8):e37547. doi: 10.2196/37547 (PMC9399884; doi:10.2196/37547)
Supplement: Multimedia Appendix 1 [file mhealth_v10i8e37547_app1.docx]

**Multimedia Appendix 1**

**Full Literature Search Strategy (adjusted for different “controlled vocabulary” used in databases)**

*(date of search - 15.04.2021.)*

| **Database** | **Search query (limited to publications after 2015)** | **Results** |
| --- | --- | --- |
| **PubMed** | (((((((("Child"[Mesh]) OR (child*)) OR ("Adolescent"[Mesh])) OR (adolescent*)) OR (teen*)) OR ("Pediatrics"[Mesh:NoExp])) OR (youth*)) AND ((((((("Fitness Trackers"[Mesh]) OR (fitness track*)) OR (physical fitness track*)) OR (activity monitor*)) OR (activity track*)) OR (wearable devic*)) OR (wearabl*))) AND (((((((("Sedentary Behavior"[Mesh]) OR (sedentar*)) OR (sedentary lifestyl*)) OR (physical inactiv*)) OR (sedentary tim*)) OR ("Rest"[Mesh])) OR ("Sitting Position"[Mesh])) OR (reclin*)) AND (2015:3000/12/12[pdat]) | **692** |
| **Scopus** | TITLE-ABS-KEY (child* OR adolescent* OR teen* OR pediatric* OR youth AND "fitness track*" OR "physical fitness track*" OR "activity monitor*" OR "activity track*" OR "wearable devic*" OR wearabl* AND "sedentary behavior" OR sedentar* OR "sedentary lifestyl*" OR "physical inactiv*" OR "sedentary tim*" OR rest* OR "sitting position" OR reclin*) AND PUBYEAR AFT 2015 | **261** |
| **SPORTDiscus with Full Text** | ( child* OR adolescent* OR teen* OR pediatric* OR youth ) AND ( "fitness track*" OR "physical fitness track*" OR "activity monitor*" OR "activity track*" OR "wearable devic*" OR wearabl* ) AND ( "sedentary behavior" OR sedentar* OR "sedentary lifestyl*" OR "physical inactiv*" OR "sedentary tim*" OR rest* OR "sitting position" OR reclin* ) | **50** |
| **ProQuest** | (child* OR adolescent* OR teen* OR pediatric* OR youth) AND ("fitness track*" OR "physical fitness track*" OR "activity monitor*" OR "activity track*" OR "wearable devic*" OR wearabl*) AND ("sedentary behavior" OR sedentar* OR "sedentary lifestyl*" OR "physical inactiv*" OR "sedentary tim*" OR rest* OR "sitting position" OR reclin*) – Additional limits – Source type: Conference Papers & Proceedings | **58** |
| **Open Access Theses and Dissertations (OATD)** | (child* OR adolescent* OR teen* OR pediatric* OR youth) AND ("fitness track*" OR "physical fitness track*" OR "activity monitor*" OR "activity track*" OR "wearable devic*" OR wearabl*) AND ("sedentary behavior" OR sedentar* OR "sedentary lifestyl*" OR "physical inactiv*" OR "sedentary tim*" OR rest* OR "sitting position" OR reclin*) AND pub_dt:[2015-01-01T00:00:00Z TO *] | **14** |
| **DART - Europe E-theses Portal** | (child* OR adolescent* OR teen* OR pediatric* OR youth) AND ("fitness track*" OR "physical fitness track*" OR "activity monitor*" OR "activity track*" OR "wearable devic*" OR wearabl*) AND ("sedentary behavior" OR sedentar* OR "sedentary lifestyl*" OR "physical inactiv*" OR "sedentary tim*" OR rest* OR "sitting position" OR reclin*) | **5** |
| **Networked Digital Library of Theses and Dissertations (NDLTD)** | (child* OR adolescent* OR teen* OR pediatric* OR youth) AND ("fitness track*" OR "physical fitness track*" OR "activity monitor*" OR "activity track*" OR "wearable devic*" OR wearabl*) AND ("sedentary behavior" OR sedentar* OR "sedentary lifestyl*" OR "physical inactiv*" OR "sedentary tim*" OR rest* OR "sitting position" OR reclin*) | **5** |
