# Supplementary material for: Accuracy and Precision of Consumer-Grade Wearable Activity Monitors for Assessing Time Spent in Sedentary Behavior in Children and Adolescents: Systematic Review
Source: JMIR Mhealth Uhealth. 2022 Aug 9;10(8):e37547. doi: 10.2196/37547 (PMC9399884; doi:10.2196/37547)
Supplement: Multimedia Appendix 2 [file mhealth_v10i8e37547_app2.docx]

**Multimedia Appendix 2**

**Leave-one-out meta-analyses**

**
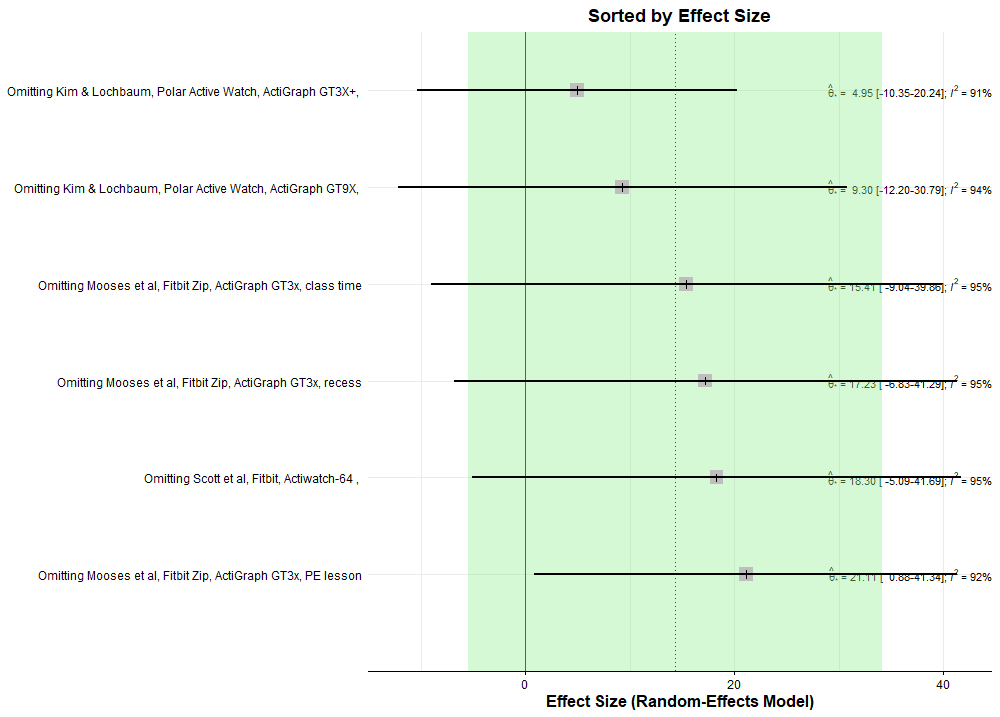
**

***Figure 1.*** Leave-one-out meta-analysis sorted by the pooled effect size

**
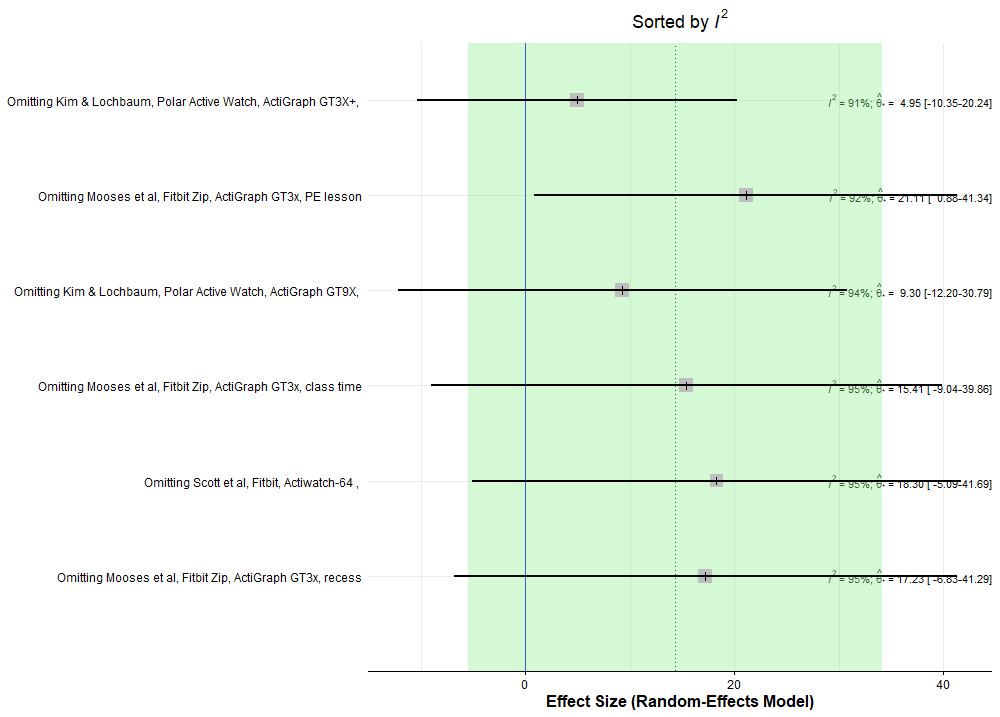
**

***Figure 2.*** Leave-one-out meta-analysis sorted by the values of I^2^ (i.e. heterogeneity)
