# Supplementary figures and images for: Accuracy and Precision of Consumer-Grade Wearable Activity Monitors for Assessing Time Spent in Sedentary Behavior in Children and Adolescents: Systematic Review
Source: JMIR Mhealth Uhealth. 2022 Aug 9;10(8):e37547. doi: 10.2196/37547 (PMC9399884; doi:10.2196/37547)

**Multimedia Appendix 3**

**Funnel plot** (*indicates a possibility of publication bias*)


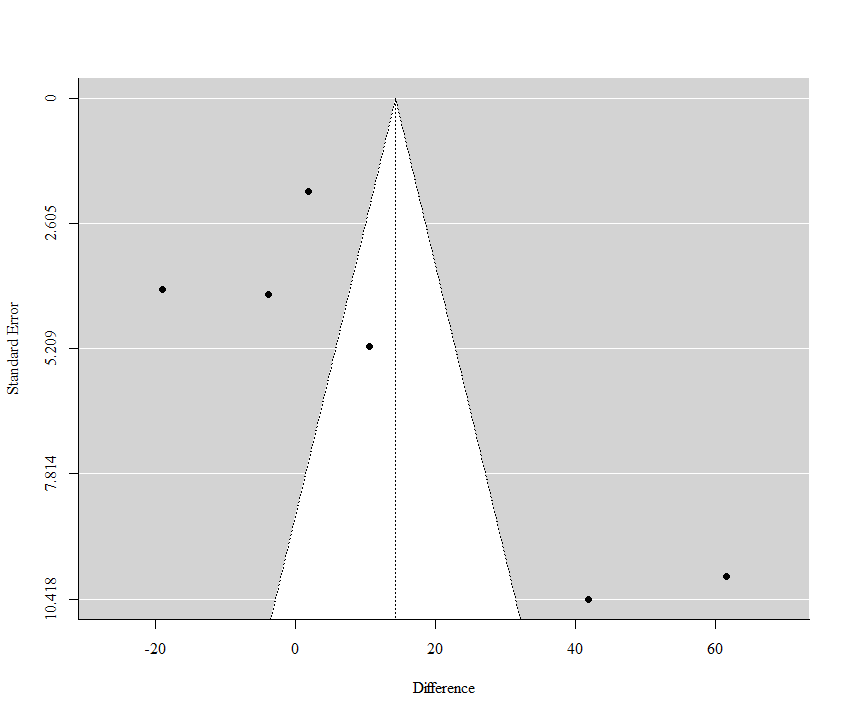

Supplement: Multimedia Appendix 3 [file mhealth_v10i8e37547_app3.docx]
